# Acquired hemophilia due to immune checkpoint inhibitors: a case series introducing emicizumab treatment

**DOI:** 10.1093/oncolo/oyaf260

**Published:** 2025-08-19

**Authors:** Louis Wolff, Carolin Ertl, Lucie Heinzerling, Sandrine Aspeslagh, Marthe Verhaert, Raphael Lattenist, Caterina Confente, Catherine Lambert, Maxime Ilzkovitz

**Affiliations:** Department of Internal Medicine, Institut Jules Bordet, Hôpital Universitaire de Bruxelles (H.U.B.), Université Libre de Bruxelles (ULB), Brussels 1070, Belgium; Belgian Multidisciplinary Immunotoxicity Board (BITOX); Department of Dermatology and Allergy, LMU University Hospital, LMU Munich, Munich 80336, Germany; Department of Dermatology and Allergy, LMU University Hospital, LMU Munich, Munich 80336, Germany; Department of Dermatology, Friedrich-Alexander-Universität Erlangen-Nürnberg (FAU), Universitätsklinikum Erlangen, Erlangen 91054, Germany; Belgian Multidisciplinary Immunotoxicity Board (BITOX); Department of Medical Oncology, Vrije Universiteit Brussel (VUB), Universitair Ziekenhuis Brussel (UZ Brussel), Brussels 1090, Belgium; Department of Internal Medicine, Vrije Universiteit Brussel (VUB), Universitair Ziekenhuis Brussel (UZ Brussel), Brussels 1090, Belgium; Belgian Multidisciplinary Immunotoxicity Board (BITOX); Department of Medical Oncology, Vrije Universiteit Brussel (VUB), Universitair Ziekenhuis Brussel (UZ Brussel), Brussels 1090, Belgium; Department of Hematology, Pôle Hospitalier Jolimont, Réseau HELORA, Haine Saint-Paul 7100, Belgium; Department of Medical Oncology, Pôle Hospitalier Jolimont, Réseau HELORA, Haine Saint‑Paul 7100, Belgium; Unité d‘hémostase-thrombose, Service d‘hématologie, Cliniques universitaires Saint-Luc, UCLouvain, Brussel 1200, Belgium; Department of Internal Medicine, Institut Jules Bordet, Hôpital Universitaire de Bruxelles (H.U.B.), Université Libre de Bruxelles (ULB), Brussels 1070, Belgium; Belgian Multidisciplinary Immunotoxicity Board (BITOX)

**Keywords:** hemophilia, immune checkpoint inhibitors, emicizumab, immunotherapy, anti-PD1, anti-PDL1, anti-CTLA4, AHA

## Abstract

**Background:**

Acquired hemophilia A (AHA) is a rare but potentially life-threatening autoimmune bleeding disorder and immune-related adverse event (irAE) associated with immune checkpoint inhibitors (ICI). This study discusses 3 new cases of ICI-induced AHA from Belgium and the SERIO-Side Effect Registry Immuno-Oncology (www.serio-registry.org), placing them in the context of existing literature.

**Methods:**

One case was encountered at a tertiary care center in Belgium. SERIO was queried and yielded another 2 cases. A comprehensive literature review using PubMed identified 8 additional cases.

**Results:**

A total of 11 patients were analyzed, with a median age of 68 years (range: 56-71), 10 were male. Most (9/11) were treated with anti-PD-1 monotherapy. In 5 cases, toxicity appeared before the fourth cycle. Immunosuppressive therapy successfully achieved complete resolution of AHA in 9 of 11 patients. For the first time, one patient was successfully treated with emicizumab, a monoclonal bispecific antibody, which bridges activated factor IX and factor X.

**Conclusions:**

Clinicians should be vigilant about ICI-induced AHA as a potentially severe irAE. Prolonged aPTT requires thorough evaluation. Upon AHA confirmation, eradication of anti-FVIII antibodies with corticosteroids, rituximab, or other immunosuppressant should be attempted. Emicizumab offers advantages over traditional replacement therapy, including ease of use and a potential reduction in immunosuppressive drug requirements—an important consideration in ICI-treated patients. Further research is needed to fully understand the pathophysiology and optimize treatment strategy for AHA.

## Background

Among the various immune-related adverse events (irAE) induced by immune checkpoint inhibitors (ICI), hematological toxicities (hem-irAE) are rare but can be life-threatening and require immediate recognition and management. These include thrombocytopenia, leucopenia and neutropenia, hemophagocytic lymphohistiocytosis, aplastic anemia, coagulation deficiency, and acquired hemophilia A (AHA).^1^ AHA is an autoimmune bleeding disorder characterized by the development of neutralizing antibodies against coagulation factor VIII and has been reported as a rare but potentially fatal hem-irAE. This study analyzed 3 new cases, one from a tertiary care center in Belgium and 2 from the SERIO Registry in the context of the already published cases of ICI-induced AHA.^2^ Interestingly, one of these cases was treated with emicizumab, a monoclonal bispecific antibody that bridges activated factor IX and factor X.^3–5^

## Methods

One case was encountered at a tertiary care center in Belgium. SERIO was queried and yielded another 2 cases. A comprehensive literature review using PubMed identified 8 additional cases.

## Results

We describe a 68-year-old man with melanoma, metastasized to the lungs and brain, treated with nivolumab and ipilimumab. He presented with subcutaneous bleeding, arms and trunk, and a prolonged activated Partial Thromboplastin Time (aPTT). Additionally work up demonstrated a FVIII < 1% with the presence of inhibitor titer at 120 BU/mL, leading to a diagnosis of acquired hemophilia (see Table 1). The patient, who was already being treated with an equivalent of prednisone 5 mg od, benefited from an increase in prednisone to 125 mg, along with rituximab and emicizumab. He benefited from the increased glucocorticoid (GC) dosage for only 7 days and from emicizumab for 3 months, as he responded rapidly both clinically and biologically to the treatment. Possibly associated with the emicizumab treatment, the increased dose of corticosteroids was only necessary for 7 days.

**Table 1.** aPCC: activated prothrombin complex concentrates.

Year	Country	Sex	Age		Cancer	ICI	Timing of toxicity	Lab results		Clinical manifestations	Immuno-suppressive treatments	Treatments to prevent bleeding	ICI	Outcome
**2011**	France	Male	42	Metastatic melanoma		Ipilimumab	After 9 weeks	aPTT 102 sec, FVIII <1%, 26 BU	Macroscopic hematuria		GC	TXA, rFVIIa	N/A	Inhibitor persisted
**2018**	Japan	Male	68	Metastatic lung squamous cell carcinoma		Nivolumab	After 17 months	aPTT 71 sec, FVIII 3%, 15 BU	Melena		GC 1 mg/kg/d, CYC	rFVIIa	Stopped	Partial remission
**2018**	Germany	Male	71	Metastatic melanoma		Pembrolizumab	After 18 weeks	N/A	N/A		RTX	rFVIIa, aPCC	N/A	Complete resolution
**2019**	USA	Male	76	Metastatic lung squamous cell carcinoma		Nivolumab	After 6 weeks	aPTT 93 sec, FVIII <1%, 31 BU	Diffuse ecchymoses, hematuria		GC, RTX	rFVIII, aPCC	Stopped	Complete resolution
**2022**	Italy	Male	67	Metastatic melanoma		Nivolumab	After 13 weeks	aPTT 99 sec, FVIII <1%, 26 BU	Macroscopic hematuria		GC 1 mg/kg/d, RTX	rFVIIa	Stopped	Complete resolution
**2022**	Italy	Male	70	Metastatic melanoma		Pembrolizumab	After 21 weeks	aPTT 70 sec, FVIII <1%, 71 BU	Diffuse ecchymoses		GC, RTX	rFVIIa	Stopped	Complete resolution
**2022**	Australia	Male	56	Metastatic lung adenocarcinoma		Atezolizumab	After 16 months	aPTT 78 sec, FVIII 4%, 14.6 BU	Anemia, bruising and hemarthrosis		GC 1 mg/kg/d, RTX	rFVIIa		Complete resolution
**2023**	Lebanon	Female	52	Metastatic lung adenocarcinoma		Not reported	After 6 weeks	aPTT 95 sec	Gynaecological bleeding		GC 1 mg/kg/d			Complete resolution
**2024**	Australia	Male	88	Invasive high-grade urothelial carcinoma		Pembrolizumab	After 3 weeks	aPTT 76 sec, FVIII 1%, 89 BU	Diffuse ecchymoses, severe anemia		GC, CYC	rFVIIa, rFVIII		Complete resolution
**2024**	Belgium	Male	68	Metastatic melanoma		Nivolumab/Ipilumab	After 12 weeks	aPTT 78, FVIII < 1%, 126 BU	Diffuse ecchymoses		GC, RTX	Emicizumab	Stopped	Complete resolution
**N/A**	Germany	Male	69	Not reported		Pembrolizumab	Not reported	Not reported	Not reported		GC 1 mg/kg/d, RTX	rFVIIA, aPCC	N/A	Complete resolution

Sources: Refs. [6–12].

Abbreviations: aPTT, activated partial thromboplastin time; BU, Bethesda Unit; CYC, cyclophosphamide; GC, glucocorticoids; rFVIIa, activated recombinant FVIIa; rFVIII, recombinant FVIII; RTX, rituximab; TXA, tranexamic Acid; N/A, Not available

With 3 new cases reported, a total of 11 patients with AHA induced by ICI with a median age of 68-year old (range: 56-71) was collected. Cancer types included 5 patients with metastatic melanoma, 4 with metastatic lung, and one with metastatic urothelial cancer. Seven patients were treated with anti-PD1, 1 with anti-PDL1, 1 with anti-CTLA4, and 1 with a combination of anti-PD1/CTLA4. In 5/11 (45%) cases, toxicity appeared before the fourth cycle. None of the patients died from a bleeding complication, even though 6 patients presented with severe AHA depicted with FVIII < 1% and inhibitor titer >20 BU/mL. All patients were treated for their toxicity with glucocorticoid (GC). Other treatments comprised cyclophosphamide (CYC), rituximab (RTX). Immunosuppression successfully achieved complete eradication of the inhibitor against FVIII in 9 out of 11 patients. One patient with recurrence of AHA was only treated with GC, the other was treated with GC and CYC (Table 1). Prevention and treatment of bleeding episodes required the use of activated prothrombin complex concentrates (aPCC), activated recombinant FVIIa (rFVIIa), recombinant FVIII (rFVIII), tranexamic acid (TXA), and emicizumab.

## Discussion

AHA is a rare auto-immune bleeding disorder characterized by the development of neutralizing antibodies against factor VIII, leading to a deficiency in factor VIII activity. We describe the largest series of ICI-induced AHA and the first case treated with emicizumab, a humanized bispecific monoclonal antibody that binds and bridges activated FIX and FX, partially mimicking FVIII activity. The pathophysiology of ICI-induced AHA is thought to involve the loss of self-tolerance and the activation of autoreactive T- and B- cells and can be induced by ICI therapy. ICI block inhibitory checkpoints on T-cells, leading to enhanced T-cell activation. This loss of self-tolerance may result in the production of autoantibodies against factor VIII, causing a deficiency in factor VIII activity and an increased risk of bleeding. The diagnosis of AHA is established by the presence of a highly prolonged aPTT, low factor VIII activity, and the detection of factor VIII inhibitors. Before concluding to an irAE, it is important to exclude other causes of AHA such as infections, other drug exposures and paraneoplastic phenomena.^13^

Treatment of acute bleeding episodes relies on hemostatic therapy while autoantibody eradication depends on immunosuppressive therapy. Corticosteroids are the first line treatment in patients with non-severe prognosis, suppressing autoantibodies in 60%-70% of the cases. Cyclophosphamide (CYC) and rituximab (RTX) are used as add-on therapy in severe cases.^13^^,^^14^ There is little evidence for second-line therapy of rare irAEs, therefore, management of ICI-induced AHA is extrapolated from non-ICI-induced AHA.

According to the latest international AHA guidelines, the use of rFVIIa, aPCC, or rpFVIII is preferred over human FVIII or desmopressin for the treatment of clinically relevant bleeding in patients with AHA.^13^

A new prophylactic hemostatic strategy with emicizumab has been developed in both congenital and acquired AHA, regardless the presence of inhibitors. This drug presents several advantages: ease of use with subcutaneous administration, activity regardless of factor VIII concentration and inhibitor’s titer, and reliability for long-term prophylaxis. It is important to note that emicizumab alters aPTT-derived coagulation parameters, and therefore, requires specific monitoring using a specialized bovine-derived test. Moreover, 2 side effects require careful monitoring during the use of emicizumab: thrombotic microangiopathy (only in case of association with high dose of aPCC). As immunosuppressive therapies carry a high risk of morbidity, including infection, using emicizumab allows to delay the use of immunosuppressive agent in the early phase of AHA diagnosis. This is of high interest in patients treated with ICI, as we are avoiding drugs that may alter the mechanisms of ICI.^1^

The question of rechallenging a patient with ICI after a severe toxicity such as AHA remains difficult. To date, no cases of rechallenge of patients with AHA have been published. It could be considered as all AHA patients survived, and nearly all had a complete resolution of the AHA. Second, a case-report of a patient with pre-existing AHA was treated with immunotherapy and did not relapse of AHA.^15^

## Conclusion

This case report highlights the rare but potentially fatal complication of AHA induced by ICI. It is crucial for clinicians to be aware of this possible irAE and, in case of clinical suspicion or unexplained aPTT elevation, to evaluate FVIII and inhibitors. Confirmed cases of ICI-induced AHA should be managed like non-ICI-induced AHA. Further research is needed to fully elucidate the pathophysiology of ICI-induced AHA and to optimize treatment and prophylaxis strategies. As a non-immunosuppressive treatment, the newly developed emicizumab appears to be a very interesting option to prevent bleeds in this type of toxicity.

## Data Availability

All data generated or analyzed during this study are included in this published article. The authors commit to providing additional information upon reasonable request.
